# Cerebral Low-Molecular Metabolites Influenced by Intestinal Microbiota: A Pilot Study

**DOI:** 10.3389/fnsys.2013.00009

**Published:** 2013-04-23

**Authors:** Mitsuharu Matsumoto, Ryoko Kibe, Takushi Ooga, Yuji Aiba, Emiko Sawaki, Yasuhiro Koga, Yoshimi Benno

**Affiliations:** ^1^Dairy Science and Technology Institute, Kyodo Milk Industry Co. Ltd.Hinode-machi, Tokyo, Japan; ^2^Benno Laboratory, RIKEN Innovation CenterWako, Saitama, Japan; ^3^Human Metabolome Technologies, Inc.Tsuruoka, Yamagata, Japan; ^4^Department of Infectious Diseases, School of Medicine, Tokai UniversityIsehara, Kanagawa, Japan

**Keywords:** intestinal microbiota, cerebrum, metabolome, gut-brain axis, neurotransmitter

## Abstract

Recent studies suggest that intestinal microbiota influences gut-brain communication. In this study, we aimed to clarify the influence of intestinal microbiota on cerebral metabolism. We analyzed the cerebral metabolome of germ-free (GF) mice and Ex-GF mice, which were inoculated with suspension of feces obtained from specific pathogen-free mice, using capillary electrophoresis with time-of-flight mass spectrometry (CE-TOFMS). CE-TOFMS identified 196 metabolites from the cerebral metabolome in both GF and Ex-GF mice. The concentrations of 38 metabolites differed significantly (*p* < 0.05) between GF and Ex-GF mice. Approximately 10 of these metabolites are known to be involved in brain function, whilst the functions of the remainder are unclear. Furthermore, we observed a novel association between cerebral glycolytic metabolism and intestinal microbiota. Our work shows that cerebral metabolites are influenced by normal intestinal microbiota through the microbiota-gut-brain axis, and indicates that normal intestinal microbiota closely connected with brain health and disease, development, attenuation, learning, memory, and behavior.

## Introduction

Intestinal microbiota play a fundamentally important role in health and diseases (Backhed et al., 2005). Recently, the relationship between intestinal microbiota and systemic phenomena beyond the intestinal environment, such as obesity (Turnbaugh et al., 2006) and lifespan (Matsumoto et al., 2011), have been reported. The bidirectional signaling between the gastrointestinal tract and the brain, the gut-brain axis, is vital for maintaining homeostasis and is regulated at the neural, hormonal, and immunological levels. The importance of the gut-brain axis is further emphasized by the high incidence of co-morbidities between stress-related psychiatric disorders such as anxiety, and gastrointestinal disorders (Camara et al., 2009). Recent studies have investigated the effect of gut microbiota on brain and behavior. The results of these studies suggest that intestinal microbiota have a great impact on gut-brain communication, which led to the coining of the term “microbiota-gut-brain axis” (MGB axis) (Rhee et al., 2009; Cryan and Dinan, 2012). For example, intestinal microbiota modulates brain development and subsequent adult behavior, such as motor activity and anxiety (Heijtz et al., 2011; Neufeld et al., 2011). Studies on the MGB axis have focused on the central nervous system (CNS), including the hypothalamic-pituitary-adrenal axis (Sudo et al., 2004; Rhee et al., 2009), neurotransmitter, and synapse related factors (for example, PSD-95, synaptophysin; Heijtz et al., 2011), and brain-derived neurotrophic factor (Heijtz et al., 2011; Neufeld et al., 2011). However, to the best of our knowledge, other metabolites stimulated by the MGB axis have not been investigated. Furthermore, some metabolites may be synthesized independently in the brain and may be influenced by MGB axis, while some metabolites produced by intestinal bacteria may be transported from the colonic lumen to the brain in the bloodstream without filtration by blood-brain barrier (BBB).

Capillary electrophoresis with time-of-flight mass spectrometry (CE-TOFMS) is a novel strategy for analyzing and differentially displaying metabolic profiles (Monton and Soga, 2007). Here, using CE-TOFMS, we analyzed the cerebral metabolome obtained from germ-free (GF) mice and Ex-GF mice, harboring intestinal microbiota from specific pathogen-free mice and demonstrated the large effect of intestinal microbiota on the cerebral metabolome.

## Materials and Methods

### Mice

Germ-free BALB/c mice were purchased originally from Japan Clea Inc. (Tokyo, Japan), and were bred in the Department of Infectious Diseases, Tokai University School of Medicine, Kanagawa, Japan. We divided six male mice bred from mating into two groups, GF mice (GF 1–3) and Ex-GF mice (Ex-GF 1–3). Mice were housed in Trexler-Type flexible film plastic isolators with sterilized clean tip (CLEA Japan, Inc., Tokyo) as bedding. They were given sterilized water and sterilized commercial CL-2 pellets, which consisted of moisture (8.5%), crude protein (24.5%), crude fat (8.0%), crude fiber (4.4%), crude ash (8.5%), and nitrogen free extracts (48.2%), corresponding to 344.7 kcal/100 g (CLEA Japan, Inc.), *ad libitum*. The diet was sterilized with an autoclave (121°C, 30 min). Surveillance for bacterial contamination was performed by periodic bacteriological examination of feces throughout the experiments. Ex-GF mice were inoculated at 4 weeks of age into the stomach by a metal catheter with 0.5 mL of a 10^−1^ suspension of feces obtained from SPF BALB/c mice. The protocols approved by the Kyodo Milk Animal Use Committee (Permit Number: 2009-02) and all experimental procedures were performed according to the guidelines of the Animal Care Committee of Tokai University.

### Specimen preparation and CE-TOFMS

Mice (7-week-old mice) were sacrificed by cervical dislocation. The brain was resected on ice, and prefrontal cortex was sliced between 2.5 and 3.5 mm anterior to bregma within 5 min of sacrifice. Immediately after the sacrifaction, cardiac blood (approximately approximately 100 μl) was collected, and sodium ethylenediamine tetraacetate plasma (final concentration was 0.13%) was prepared by centrifugation for 20 min at 2,300 × *g* and 4°C. The samples were stored at −80°C until use.

Cardiac plasma (50 μl) and methanol (450 μl) with 50 μM intestinal standard were vortexed. The plasma homogenate served as crude metabolome and was added to chloroform (500 μl) and Milli-Q (200 μl), mixed, and centrifuged (2,300 × *g*, for 5 min at 4°C). The aqueous layer was centrifugally filtered through a 5-kDa cutoff filter Ultrafree-MC (Millipore). The filtrated solution was dried up and suspended in 25 μL Milli-Q water just before the measurement. The cerebrums were suspended in methanol (500 μl) with 50 μM intestinal standard and vortexed vigorously five times for 60 s with a MicroSmash MS-100R (Tomy Digital Biology Co., Ltd., Tokyo, Japan) at 4,000 rpm. The resulting cerebrum sample served as crude metabolome that subsequently underwent the same treatment as the plasma crude metabolome.

Metabolomics measurement and data processing were performed as described previously with an Agilent Capillary Electrophoresis System (Ooga et al., 2011). The CE-MS system is the Agilent G1600A Capillary Electrophoresis System connected with the Agilent G1969A LC/MSD TOF (Agilent Technologies, Palo Alto, CA, USA).

### RNA preparation and quantitative real-time PCR of the cerebrums

Frozen prefrontal cerebrums were processed for total RNA preparation with TaKaRa FastPure RNA Kits (Takara Bio Inc., Otsu, Japan). The quantity, purity, and integrity were confirmed initially by electrophoresis. cDNA for each sample was synthesized using 200 ng total RNA and PrimeScript RT reagent Kits (Takara Bio Inc.). Real-time PCR was performed with a StepOne Real-Time PCR System (Applied Biosystems) with TaqMan Fast Universal PCR Master Mix (Applied Biosystems) using TaqMan probes (hexokinase 1: Mm00439344_m1, phosphofructokinase: Mm00445461_m1, and β-actin: Mm02619580_g1). The comparative delta *C*_t_ method was used for normalizations to the housekeeping gene β-actin.

### Intestinal bacterial compositions

Bacterial compositions were determined using pyrosequencing system. Bacterial DNA was isolated from colonic content samples of mice. The 16S rRNA was targeted to identify intestinal bacteria and a pair of universal primers; 27f (5 –AGA GTT TGA TCC TGG CTC AG–3) and 350r (5 –CTG CTG CCT CCC GTA G–3) were used for PCR. Amplicons were applied to GS titanium sequencing Kit (Roche Diagnostics) include emulsion PCR and analyzed by Genome sequencer FLX system (Roche Diagnostics). About 18,000–20,500 sequences in each sample were identified. Sequences data were compared with DDBJ database (Blast) and classified by taxonomic categories.

### Data analysis and statistics

Clustering analysis in metabolome was processed by MATLAB 2008a (MathWorks, MA, USA). Differences in relative quantity between GF mice and Ex-GF mice were evaluated for individual metabolites by Welch’s *t*-test.

## Results

### The difference in cerebral metabolome between GF and Ex-GF mice

**Figure 1:**
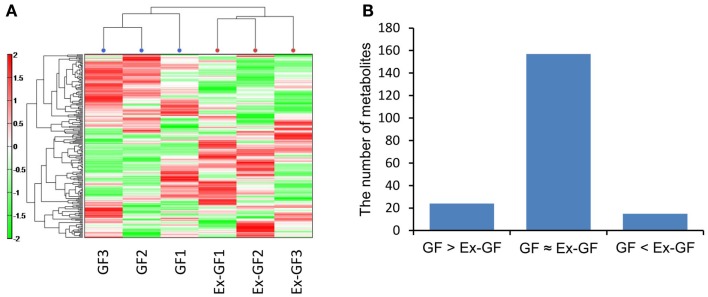
**Difference in the cerebral metabolome between GF mice and Ex-GF mice**. **(A)** Hierarchical clustering showing patterns of metabolites. Red and green indicate high and low concentrations of metabolites, respectively. **(B)** The number of cerebral metabolites in the group. GF > Ex-GF; GF ≈ Ex-GF; and GF < Ex-GF.

When the mice were sacrificed, the body weights of GF mice were between 22 and 24 g and those of Ex-GF mice were between 22 and 25 g. CE-TOFMS identified 196 (120 cations and 76 anions) metabolites from the cerebral metabolome in both of GF and Ex-GF mice. Hierarchical clustering of metabolite patterns is shown in Figure 1A. A remarkable difference was observed in the cerebral metabolome between GF and Ex-GF mice. Of the 196 metabolites in the cerebral metabolome, the concentrations of 23 metabolites were at least 1.6-fold, and/or significantly (*p* < 0.05) higher, in GF mice than Ex-GF mice (group GF > Ex-GF). A further 15 metabolites were at least 1.6-fold, and significantly (*p* < 0.05) higher, in Ex-GF mice than GF mice (group GF < Ex-GF), and/or 158 metabolites showed no difference in concentration or incidence between GF and Ex-GF mice (Figure 1B).

Identified metabolites were classified into eight categories and are listed in Table A1 in Appendix (anion) and Table A2 (cation) in Appendix. Metabolites, in which there are significant differences between GF and Ex-GF mice, are shown in Tables 1 and 2.

**Table 1:** **Metabolites whose concentrations were higher in the cerebral metabolome of Ex-GF mice than in that of GF mice**.

Compound name	Category	Mean		SD		Ratio	
		GF	Ex-GF	GF	Ex-GF	Ex-GF/GF	
Trimethylamine *N*-oxide	Alkylamino acid	1.87E-05	8.20E-05	3.37E-06	1.53E-05	4.39	*
*N*^5^-Ethylglutamine	Alkylamino acid	6.06E-05	1.43E-04	6.62E-06	2.43E-05	2.36	*
Cysteine glutathione disulfide	Peptide	3.12E-04	6.78E-04	2.78E-04	4.36E-04	2.17	
2,3-Diphosphoglyceric acid		8.67E-05	1.61E-04	2.73E-05	3.98E-05	1.85	*p* < 0.1
Cys	Amino acid	8.61E-04	1.54E-03	7.09E-04	9.51E-04	1.79	
2-Methylserine		4.90E-05	8.70E-05	4.25E-06	1.25E-05	1.78	*
3-Methylhistidine	Alkylamino acid	6.14E-04	1.03E-03	6.71E-05	1.34E-04	1.68	*
Cystine	Peptide	1.96E-05	3.28E-05	NA	5.53E-06	1.67	
Trp	Amino acid	9.74E-04	1.44E-03	2.74E-05	1.13E-04	1.48	*
Pipecolic acid		1.61E-04	2.33E-04	7.37E-07	1.28E-05	1.44	*
Tyr	Amino acid	3.47E-03	4.75E-03	3.16E-04	3.81E-04	1.37	*
Phe	Amino acid	3.74E-03	4.97E-03	1.44E-04	1.38E-04	1.33	***
Asp	Amino acid	2.72E-03	3.43E-03	2.78E-05	1.20E-04	1.26	**
Ribose 5-phosphate	Energy	1.11E-04	1.32E-04	3.22E-06	9.09E-06	1.19	*
Gln	Amino acid	7.12E-03	8.46E-03	1.74E-04	4.57E-04	1.19	*

*p < 0.05, **p < 0.01, ***p < 0.001 (GF vs. Ex-GF).

*These metabolites have significant or more than 1.6-fold difference between GF mice and Ex-GF*.

**Table 2:** **Metabolites whose concentrations were lower in the cerebral metabolome of Ex-GF mice than in that of GF mice**.

Compound name	Category	Mean		SD		Ratio	
		GF	Ex-GF	GF	Ex-GF	Ex-GF/GF	
*N*-Acetylneuraminic acid	Alkylamino acid	1.03E-03	8.75E-04	4.35E-05	5.59E-05	0.85	*
*N*-Acetylaspartic acid	Neuron transmitter	2.06E-01	1.72E-01	1.04E-02	8.54E-03	0.84	*
Pantothenic acid	Co-enzyme	4.37E-04	3.58E-04	2.08E-05	3.18E-05	0.82	*
Biotin	Co-enzyme	2.22E-04	1.79E-04	1.66E-05	1.83E-05	0.80	*
Ser	Amino acid	1.05E-03	7.95E-04	6.49E-05	5.53E-05	0.76	**
ADP	Nucleic acid	5.09E-03	3.85E-03	5.37E-04	1.32E-04	0.76	*
1-Methylnicotinamide	Alkylamino acid	5.04E-05	3.71E-05	5.17E-06	5.00E-06	0.74	*
Ser-Glu	Peptide	4.69E-05	3.42E-05	5.47E-06	4.85E-06	0.73	*
Succinic acid	Energy	2.01E-02	1.45E-02	1.25E-03	1.68E-03	0.72	*
3-Phenylpropionic acid		1.23E-04	8.80E-05	1.30E-05	9.03E-06	0.71	*
Dihydroxyacetone phosphate	Energy	2.02E-04	1.40E-04	1.23E-05	2.27E-05	0.69	*
IMP	Nucleic acid	2.67E-03	1.84E-03	1.70E-04	2.63E-04	0.69	*
2-Hydroxybutyric acid		8.39E-05	5.77E-05	8.91E-06	8.28E-06	0.69	*
NADP^+^	Co-enzyme	8.69E-05	5.68E-05	3.79E-06	1.27E-05	0.65	*
Hydroxyproline	Amino acid	1.64E-03	1.07E-03	3.18E-05	1.14E-04	0.65	**
NADH	Co-enzyme	2.09E-04	1.35E-04	2.26E-05	2.52E-05	0.65	*
3-Phosphoglyceric acid		5.54E-04	3.44E-04	9.54E-05	2.31E-05	0.62	*p* < 0.1
Glycerol 3-phosphate		3.77E-03	2.31E-03	2.93E-04	1.27E-03	0.61	
Glucose 6-phosphate	Energy	1.01E-04	6.03E-05	2.05E-05	2.93E-06	0.60	*p* < 0.1
Fructose 6-phosphate	Energy	2.68E-05	1.54E-05	7.92E-06	2.03E-06	0.57	
Dopamine	Neuron transmitter	5.26E-04	2.85E-04	2.16E-04	5.60E-05	0.54	
Fructose 1,6-diphosphate	Energy	5.43E-04	2.67E-04	1.52E-04	4.13E-05	0.49	*p* < 0.1
Taurocholic acid	Bile acid	1.51E-04	2.26E-05	3.08E-05	1.17E-05	0.15	**

*p < 0.05, **p < 0.01, ***p < 0.001 (GF vs. Ex-GF).

*These metabolites have significant or more than 1.6-fold difference between GF mice and Ex-GF*.

### Influence of intestinal microbiota on cerebral glycolytic metabolism

**Figure 2:**
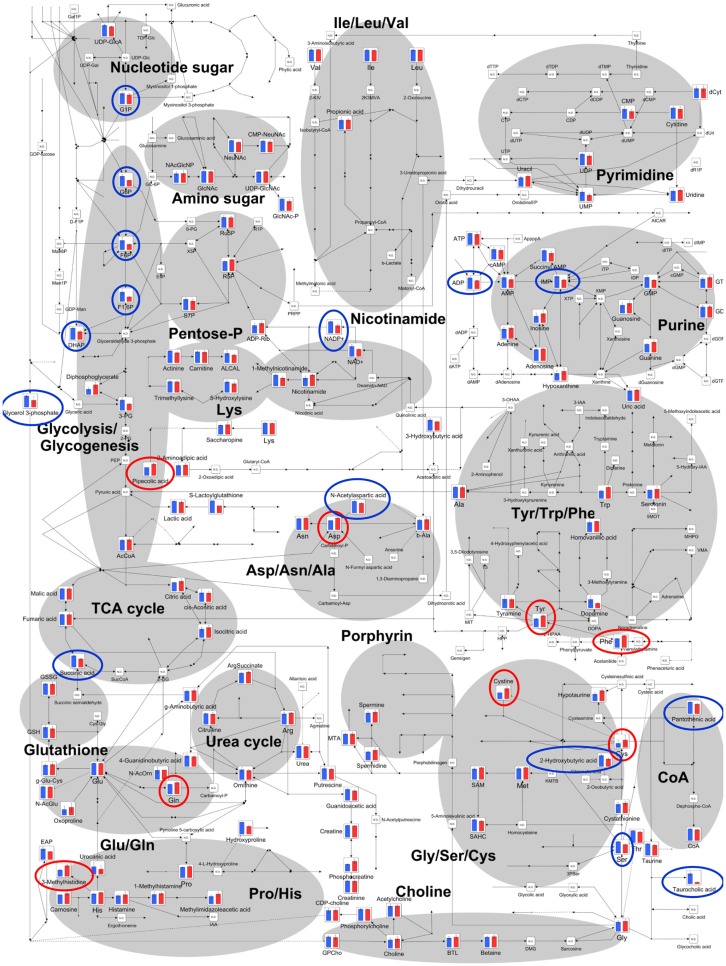
**Differences of cerebral metabolites between GF mice and Ex-GF mice on the principal metabolic pathways**. The relative quantities of the annotated metabolites are represented as bar graphs (blue, GF: red, Ex-GF). Metabolites surrounded by blue and red circles are of higher and lower concentrations, respectively, in GF mice than Ex-GF mice. ND, not detected.

**Figure 3:**
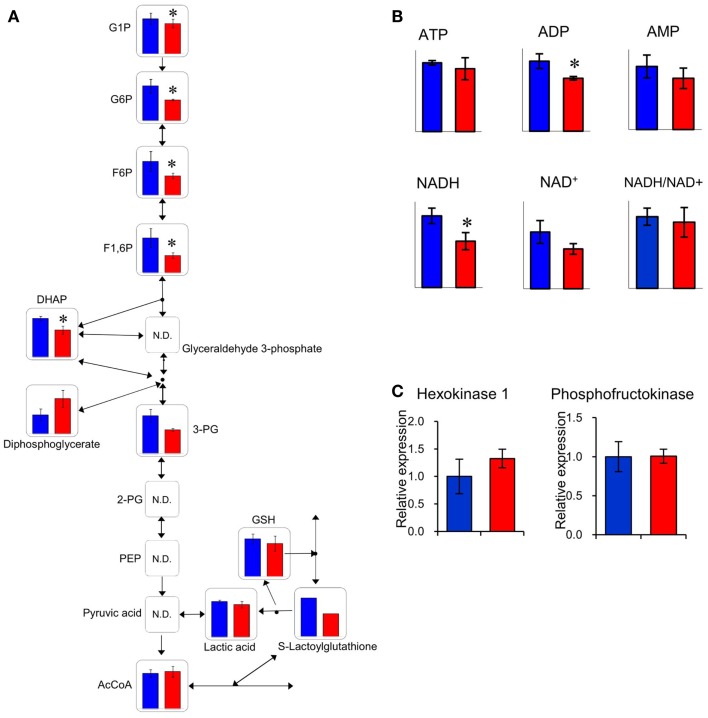
**Comparison of glycolytic metabolic activity between GF mice and Ex-GF mice**. **(A)** The relative quantities of the annotated metabolites are represented as bar graphs (blue, GF: red, Ex-GF). **(B)** Relative quantities of ATP, ADP, AMP, and nicotinamides (*p < 0.05). **(C)** Cerebral gene expression of hexokinase and phosphofructokinase. Data are represented as mean ± SD **(A,B)** and mean ± SEM **(C)**.

The relative quantities of the annotated metabolites in the principal metabolic pathways are represented as bar graphs (Figure 2). The concentrations of metabolites involved in glycolysis/gluconeogenesis pathways are characteristically higher in GF mice than in Ex-GF mice. Therefore, we focused our work on cerebral glycolytic metabolism (Figure 3A). The concentration of ADP and NADH were significantly (*p* < 0.05) higher, while there was a tendency for concentrations of ATP, AMP, and NAD^+^ to be higher in GF mice than Ex-GF mice. The NADH/NAD^+^ ratio tended to be lower in GF mice than in Ex-GF mice (Figure 3B). There was no difference in the expression of the hexokinase and phosphofructokinase genes, between GF mice and Ex-GF mice (Figure 3C).

### Comparison of individual differences in metabolome between colonic luminal content, cardiac plasma, and the cerebrum

**Figure 4:**
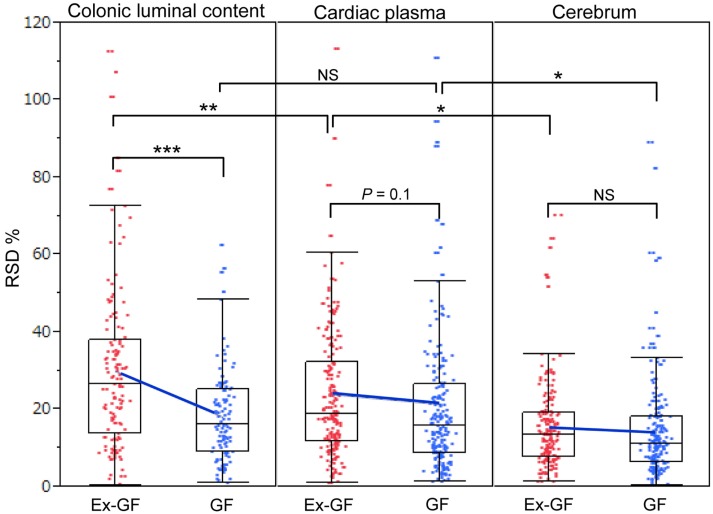
**The boxplot of RSD% of all metabolites detected from colonic luminal content, cardiac plasma, and the cerebrum**. Blue cross bars represent the comparison of means between Ex-GF mice and GF mice. *p < 0.05, **p < 0.01, ***p < 0.001 (GF vs. Ex-GF).

Relative standard deviations (RSD% = value of standard deviation/value of mean × 100) of metabolites in the colonic luminal content, cardiac plasma, and the cerebrum of GF and Ex-GF mice are shown in Figure 4. The RSD value of metabolites in the cerebrum was similar between GF and Ex-GF mice. However, in Ex-GF mice, the RSD values in cardiac plasma (*p* = 0.10) and colonic luminal content (*p* < 0.001) were larger than in GF mice. In addition, in Ex-GF mice, the RSD values were the highest for colonic content (vs. cardiac plasma, *p* < 0.01), followed by cardiac plasma (vs. the cerebrum, *p* < 0.05) and the cerebrum. In contrast, in GF mice, the RSD value did not differ between colonic content and cardiac plasma, although that of cardiac plasma was greater than that of the cerebrum (*p* < 0.05).

### Comparisons of metabolites between colonic luminal content, cardiac plasma, and the cerebrum

**Figure 5:**
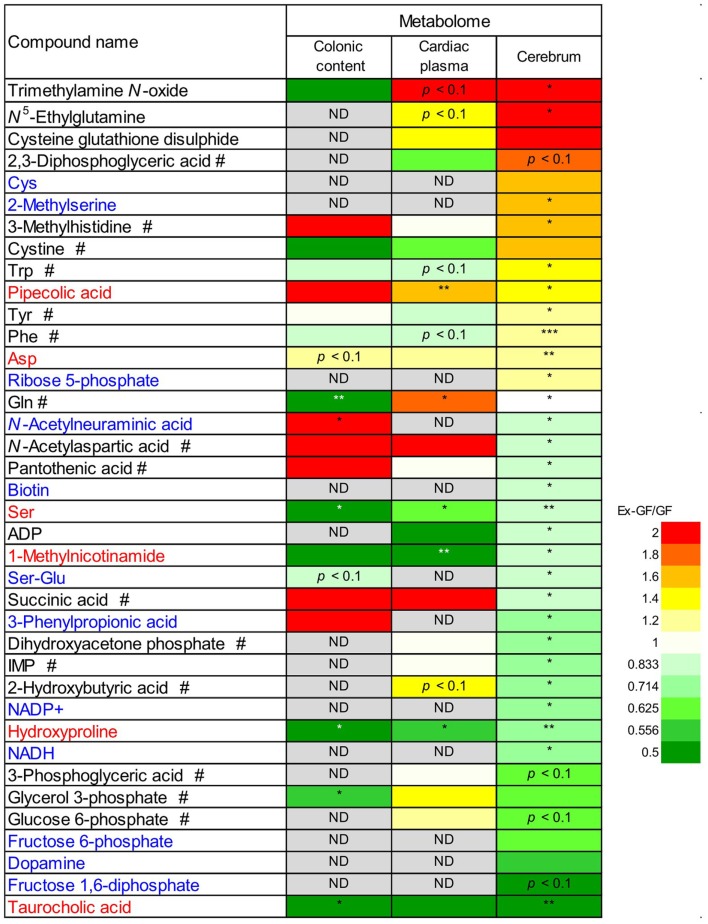
**Relative quantitative ratio (Ex-GF/GF value) comparisons of 38 metabolites between GF mice and Ex-GF mice, colonic luminal content, cardiac plasma, and the cerebrum**. Metabolites shown in red have similar Ex-GF/GF ratios between the colonic lumen, cardiac plasma, and the cerebrum. Metabolites shown in blue are below the detection limit in cardiac plasma, but were detected in the cerebrum. ^#^These metabolites differed in Ex-GF/GF ratios between the cerebrum and cardiac plasma. *p < 0.05, **p < 0.01, ***p < 0.001 (GF vs. Ex-GF). ND, not detected.

We compared the 38 metabolites, which were significantly altered between the cerebrum of GF and Ex-GF mice. The relative quantitative ratio (Ex-GF/GF value) for the expression of each metabolite in colonic luminal content, cardiac plasma, and the cerebrum are shown in Figure 5. Six metabolites, which are shown in red, had similar Ex-GF/GF ratios in all three sites. Although detected in the cerebrum, 12 metabolites, which are shown in blue, were below the detection limit in cardiac plasma. A total of 16 metabolites, which are marked by the “#” symbol, had different Ex-GF/GF ratios between the cerebrum and cardiac plasma. The Ex-GF/GF ratios of all other metabolites did not differ between the three specimens.

### Intestinal bacterial compositions

**Figure 6:**
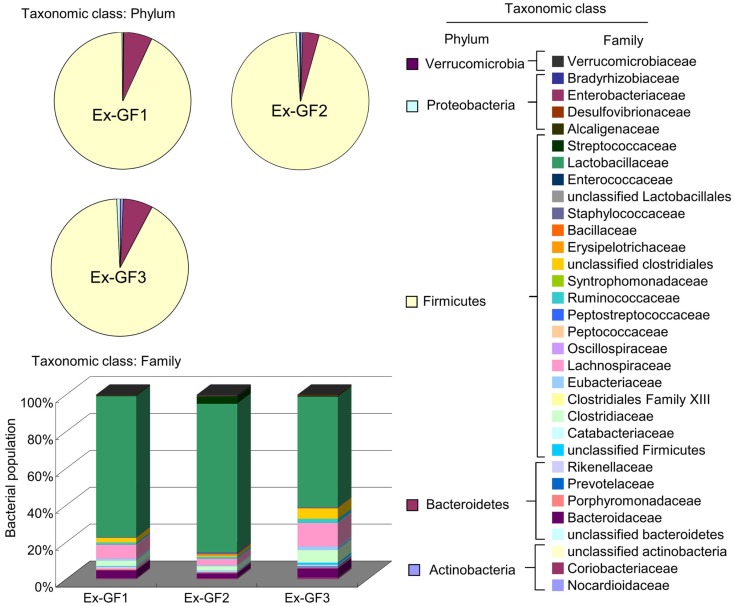
**Aggregate microbiota composition at the phylum and family levels in the colonic content of Ex-GF mice**.

Bacterial compositions were analyzed using FLX systems and the results are shown in Figure 6. Phylum Firmicutes (80%) and phylum Bacteroidetes (about 6%) have been identified as dominant populations in all samples. Following detailed classification on the family level, the families *Lactobacillaceae*, *Lachnospiraceae*, *Clostridiaceae*, and *Bacteroidaceae* were commonly detected and constituted higher proportions in the population, i.e., 50–70, 3–10, 2–5, and 2–4% respectively, than other families. However, there were only small individual differences among the samples. These families accounted for up to 60–70% of the total bacterial population.

## Discussion

To the best of our knowledge, in a prior study by Fu et al. (2011), the highest numbers of metabolites from brain tissue to date were detected using GC-MS. In total, 118 metabolites were routinely detected in more than 80% of samples in one or more of three species (human, chimpanzee, or rhesus macaques), in at least one brain region (prefrontal or cerebellar cortex). However, only 61 metabolites were annotated. CE-TOFMS identified 196 metabolites from the cerebral metabolome, indicating that CE-TOFMS is more sensitive than GC-MS for comprehensive and large-scale metabolomic analysis in the brain.

### Neurotransmitters and several metabolites which are involved in brain function

**Figure 7:**
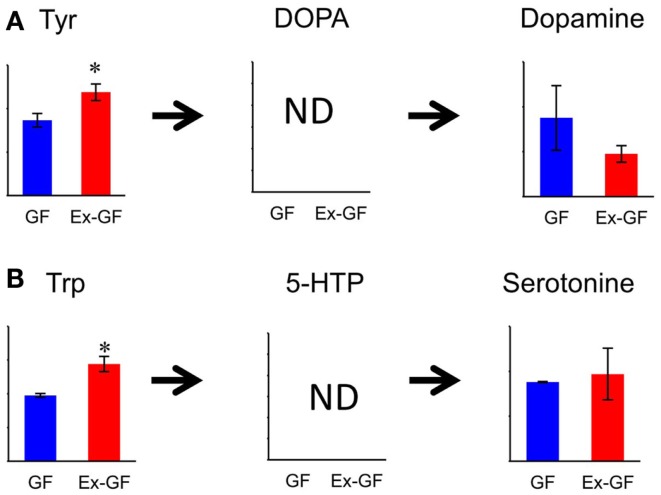
**Relative quantitative comparisons of metabolites in the biosynthetic pathway for dopamine (A) and serotonin (B), in the cerebrum of GF mice and Ex-GF mice**. Data are represented as mean ± SD. *p < 0.05, ***p < 0.001 (GF vs. Ex-GF).

Concentration of dopamine (DA), a target for amphetamine stimulation of locomotor activity and stereotyped behaviors, was approximately twofold higher (*p* = 0.188) in GF mice than in Ex-GF mice. This is consistent with the findings that GF mice display increased motor activity and reduced anxiety compared with their Ex-CF counterparts (Heijtz et al., 2011; Neufeld et al., 2011). It is confusing that the concentration of Tyr in the cerebrum of Ex-GF mice was higher than that of GF mice, since Tyr is a precursor of DA. Tyr hydroxylase hydroxylates Tyr to l-DOPA, which was below the detection limit in this study. DOPA is further converted to DA by aromatic amino acid decarboxylase (Daubner et al., 2011). Therefore, this indicates that cerebral DA synthesis is induced by DA-producing enzymes, which are inhibited by stimulation of intestinal microbiota through the MGB axis (Figure 7A). Parkinson disease is characterized by a progressive loss of dopaminergic neurons in the substantia nigra. Since the activity level of Tyr hydroxylase is associated with Parkinson disease (Haavik and Toska, 1998), it is possible that the intestinal microbiota is involved in the development of Parkinson disease.

We were also surprised to find that the concentrations of Trp, precursors of serotonin (5-HT), in the cerebrum of Ex-GF mice were higher than that of GF mice. This was despite the fact that cerebral 5-HT concentration did not differ between GF mice and Ex-GF mice (Figure 7B). It is believed that brain 5-HT concentration is dependent on the brain Trp level (Fernstrom, 2005). Plasma Trp are transported into the brain by a transporter, located at BBB on CNS capillary endothelial cells (Pardridge, 1998), and converted to 5-HT in neurons containing Trp hydroxylase, the rate-limiting enzyme in 5-HT synthesis (Jequier et al., 1967). Therefore, we suppose that cerebral 5-HT synthesis is regulated by Trp hydroxylase in neurons without the influence of the cerebral Trp pool and/or intestinal microbiota under the non-stressed condition and in non-neonates, as in our present study.

Several metabolites, which are known to be involved in brain function, are also influenced by normal intestinal microbiota. *N*-acetylaspartic acid (NAA), which is in group GF > Ex-GF, is an amino acid present in the vertebrate brain that is synthesized and stored primarily in neurons and considered a marker for neuronal health and attenuation (Simmons et al., 1991; Jenkins et al., 2000). Pipecolic acid, which is in the GF < Ex-GF group, is known as a neuromodulator or neurotransmitter with the gamma-aminobutyric acid (GABA)ergic transmission. Pipecolic acid was shown to be region- and site-specific in the CNS (Kase et al., 1980), which causes hepatic encephalopathy by inducing neuronal cell death, or apoptosis, rather than by depressing neurotransmissions (Matsumoto et al., 2003). Ser was in the GF > Ex-GF group; d-Ser is synthesized from l-Ser by serine racemase (CE-TOFMS could not separate d-Ser and l-Ser) in the human brain. It functions as an obligatory co-agonist at the glycine modulatory site of *N*-methyl-d-aspartate (NMDA)-selective glutamate receptors. Thus, depletion of d-Ser levels has been implicated in NMDA receptor hypofunction, which is thought to occur in schizophrenia (Yang et al., 2010). *N*-acetylneuraminic acid (NANA), which was in group GF > Ex-GF, increased learning and memory performance (Wang et al., 2007). These findings indicate that intestinal microbiota are closely related to brain health, disease development, attenuation, learning, and memory.

### Cerebral energy metabolism

The concentration of several cerebral glycolysis intermediates was higher in GF mice than in Ex-GF mice (Figure 3A). This raises the following two possibilities: first, the cerebral energy consumption of Ex-GF mice is higher than that of GF mice, and second, that cerebral energy production by glycolysis in Ex-GF mice is lower than in GF mice. However, these phenomena presumably indicate an accelerated molecular flux of the glycolysis pathway to compensate for ATP and NADH depletion in the cerebrum of Ex-GF mice. This assumption is based on our finding that the cerebral ATP (Ex-GF/GF ratio = 0.91) and NADH (Ex-GF/GF ratio = 0.65) levels were lower in Ex-GF mice than GF mice (Figure 3B) and there was no difference in cerebral hexokinase and phosphofructokinase gene expression between mice (Figure 3C). In fact, levels of acetyl CoA, which is produced by oxidation from pyruvic acid, was similar in the cerebrum of GF and Ex-GF mice. Furthermore, a significant difference in lactic acid was not observed, suggesting that the normal intestinal microbiota do not influence anaerobic respiration and the compensated molecular components (ATP or NADH) of the glycolysis pathway in Ex-GF mice was then transferred into the TCA cycle for further aerobic respiration via acetyl CoA in the cerebral mitochondria. To support the presence of an active TCA cycle, we also report changes in NADH and NAD^+^. The ratio between NADH and NAD^+^ affects mitochondrial TCA cycle activity (LaNoue et al., 1972). NADH and NADH/NAD^+^ ratio in Ex-GF mice were reduced to 65 and 92% of those in GF mice, respectively. Since both values are known to increase when the TCA cycle is blocked (Sugiura et al., 2011), the observed reductions in NADH and NADH/NAD^+^ ratio suggest normal intestinal microbiota induces active oxidative phosphorylation via the TCA cycle. From these findings, we suggest that, in the cerebrum, Ex-GF mice consume energy and accelerate energy production through glycolysis and TCA cycle more highly than GF mice. In other word, the cerebrum of Ex-GF mice is more active than that of GF mice.

### Bacterial potential influence on cerebrum metabolic changes

**Figure 8:**
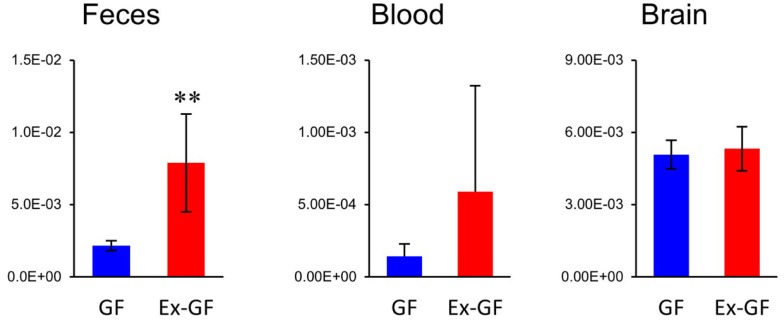
**Relative quantitative comparisons of GABA in colonic lumen content, cardiac plasma, and the cerebrum**. Data are represented as mean ± SD. **p < 0.01.

Of 38 metabolites influenced by intestinal microbiota, 12 metabolites detected from the cerebrum but not cardiac plasma, are synthesized independently in the cerebrum and are influenced by MGB axis (Figure 5, metabolites shown in blue). Sixteen metabolites whose Ex-GF/GF ratio differed between the cerebrum and cardiac plasma are influenced by MGB axis and/or BBB (Figure 5, metabolites marked by #). The fact that NANA is in this group is in conflict existing literature. In animal infant models, exogenous administration of NANA increased learning and memory performance as well as the concentration of NANA in the frontal cortex (Carlson and House, 1986; Wang et al., 2007). However, in the present study, NANA produced by intestinal microbiota was not transported to the blood. Therefore, it is doubtful whether dietary NANA influences the brain and behavior directly. We suppose that improvement of learning and memory performance by oral administration of NANA depends on the stimulation of intestinal microbiota, which is altered by supplements containing NANA through the MBG axis. Cerebral GABA concentration did not differ between GF mice and Ex-GF mice, although remarkable differences were observed in GABA cardiac plasma concentrations between GF mice and Ex-GF mice (Figure 8). This indicates that GABA is controlled by BBB and tightly regulated in the cerebrum. This questions the suitability of oral GABA supplementation studies to provide GABA to the brain.

Differences in RSD values between GF mice and Ex-GF mice (Figure 4) implies that individual differences in the metabolites found in the colonic content and cardiac plasma of Ex-GF mice is influenced by the diversity of intestinal microbiota (Matsumoto et al., 2012). Furthermore, these findings indicate that many metabolites produced by intestinal bacteria are filtrated and transported to brain via the blood through the BBB. However, six cerebral metabolites (Figure 5, metabolites shown in red) had similar Ex-GF/GF ratios between colonic luminal content, cardiac plasma, and the cerebrum. This may suggest that these metabolites may be transported from the colonic lumen to the cerebrum in the bloodstream without filtration by BBB. Further studies are required to fully understand how these metabolites are transported from the gut lumen to blood and from blood to the brain. Furthermore, the relationship between intestinal bacterial composition and brain metabolome is an area that clearly merits further study in the future.

These discussions center on a comparison between general knowledge and the data obtained in the present study. However, the neuronal effects of almost detected metabolites in the cerebrum are unclear. In future studies, researchers in various fields may find evidence that some of the newly identified metabolites are important for neuronal activities and diseases. Indeed, there is a possibility of detecting site-specific metabolome profiles when using CE-TOFMS. Further studies are required to analyze other parts of the brain.

In this study, many metabolites including neurotransmitters showed differences in the concentrations between GF mice and Ex-GF mice, indicating that normal intestinal microbiota closely connected with brain health and disease, development, attenuation, learning, memory, and behavior. We propose that through proper control of intestinal microbiota, cerebral nerve disorders may be prevented or alleviated in the future.

## Conflict of Interest Statement

This work was supported by the BRAIN, Japan. This work was also funded by Kyodo Milk Industry Co. Ltd and Human Metabolome Technologies, Inc. The funders had no role in study design, data collection and analysis, decision to publish, or preparation of the manuscript. Mitsuharu Matsumoto and Emiko Sawaki are employes of Kyodo Milk Industry Co. Ltd. and had a role in study design, data analysis, preparation of the manuscript, and decision to publish the manuscript. Takushi Ooga is employe of Human Metabolome Technologies, Inc. and had a role in data analysis and decision to publish the manuscript. All of the other authors declare that they have no conflict of interest.

## Author Contributions

Mitsuharu Matsumoto wrote the paper. Mitsuharu Matsumoto, Yasuhiro Koga, and Yoshimi Benno designed this study. Yuji Aiba performed animal experiments. Takushi Ooga analyzed the metabolome. Mitsuharu Matsumoto, Ryoko Kibe, Takushi Ooga, and Emiko Sawaki analyzed the data, discussed findings, and helped draft the manuscript.

## Appendix

**Table A1:** **Anionic metabolites detected from cerebrum in GF mice and Ex-GF mice**.

ID	HMT DB^†^			Relative area^§^										Comparative analysis		
	Compound name	KEGG ID	HMDB ID	Germ-free			SPF			Germ-free		SPF		SPF vs. Germ-free		
				GF1	GF2	GF3	Ex-GF1	Ex-GF2	Ex-GF3	Mean	SD	Mean	SD	Ratio^1^	*p*-Value^2^	
A_0052	2,3-Diphosphoglyceric acid	C01159	HMDB01294	7.6E-05	6.7E-05	1.2E-04	1.5E-04	1.3E-04	2.0E-04	8.7E-05	2.7E-05	1.6E-04	4.0E-05	1.853	0.064	
A_0007	2-Hydroxyisobutyric acid	–	HMDB00729	1.3E-04	1.6E-04	1.8E-04	3.0E-04	2.2E-04	2.1E-04	1.6E-04	2.3E-05	2.4E-04	4.7E-05	1.525	0.073	
A_0041	Phosphocreatine	C02305	HMDB01511	1.1E-04	1.1E-04	9.9E-05	1.8E-04	1.6E-04	1.1E-04	1.1E-04	7.2E-06	1.5E-04	3.5E-05	1.430	0.146	
A_0015	5-Oxoproline	C01879	HMDB00267	1.9E-03	1.0E-03	5.4E-04	2.5E-03	1.6E-03	7.5E-04	1.1E-03	6.9E-04	1.6E-03	8.6E-04	1.398	0.517	
A_0088	ADP-ribose	C00301	HMDB01178	2.2E-04	2.3E-04	3.8E-04	2.6E-04	3.8E-04	4.1E-04	2.7E-04	9.0E-05	3.5E-04	7.8E-05	1.272	0.338	
A_0044	Ribose 5-phosphate	C00117	HMDB01548	1.1E-04	1.1E-04	1.1E-04	1.3E-04	1.4E-04	1.2E-04	1.1E-04	3.2E-06	1.3E-04	9.1E-06	1.190	0.044	*
A_0037	Isocitric acid	C00311	HMDB00193	1.1E-04	1.7E-04	1.2E-04	1.3E-04	1.5E-04	1.9E-04	1.3E-04	3.1E-05	1.6E-04	3.1E-05	1.189	0.377	
A_0087	GTP	C00044	HMDB01273	4.1E-04	3.9E-04	5.3E-04	6.2E-04	4.7E-04	4.6E-04	4.5E-04	7.9E-05	5.2E-04	9.0E-05	1.167	0.345	
A_0067	NADPH_divalent	C00005	HMDB00221	5.4E-05	7.7E-05	7.4E-05	8.5E-05	6.9E-05	8.5E-05	6.8E-05	1.2E-05	8.0E-05	9.4E-06	1.166	0.281	
A_0013	2-Hydroxyvaleric acid	–	HMDB01863	1.5E-04	1.7E-04	1.1E-04	1.9E-04	1.3E-04	1.9E-04	1.5E-04	2.8E-05	1.7E-04	3.3E-05	1.162	0.397	
A_0036	Citric acid	C00158	HMDB00094	6.3E-03	7.2E-03	6.3E-03	9.3E-03	5.7E-03	7.8E-03	6.6E-03	5.3E-04	7.6E-03	1.8E-03	1.153	0.433	
A_0032	Homovanillic acid	C05582	HMDB00118	9.4E-05	1.6E-04	1.6E-04	1.5E-04	1.6E-04	1.5E-04	1.4E-04	3.8E-05	1.5E-04	5.1E-06	1.109	0.568	
A_0048	*myo*-Inositol 1-phosphate *myo*-Inositol 3-phosphate	C01177 C04006	HMDB00213 HMDB06814	6.6E-04	6.0E-04	5.5E-04	7.3E-04	5.9E-04	6.7E-04	6.0E-04	5.8E-05	6.6E-04	6.7E-05	1.098	0.317
A_0025	Uric acid	C00366	HMDB00289	6.0E-05	7.8E-05	5.9E-05	6.3E-05	7.8E-05	6.9E-05	6.6E-05	1.1E-05	7.0E-05	7.9E-06	1.068	0.592	
A_0074	Acetyl CoA_divalent	C00024	HMDB01206	5.5E-05	5.4E-05	4.6E-05	4.6E-05	5.6E-05	6.1E-05	5.2E-05	5.3E-06	5.4E-05	7.9E-06	1.048	0.677	
A_0023	3-Hydroxy-3-methylglutaric acid	C03761	-	3.5E-04	3.1E-04	3.0E-04	3.6E-04	3.4E-04	3.1E-04	3.2E-04	2.3E-05	3.4E-04	2.4E-05	1.047	0.479	
A_0077	GDP	C00035	HMDB01201	1.5E-03	1.7E-03	2.1E-03	1.9E-03	1.6E-03	2.0E-03	1.8E-03	3.1E-04	1.8E-03	2.3E-04	1.033	0.803	
A_0093	UDP-*N*-acetyl glucosamine	C00043	HMDB00290	9.1E-04	1.0E-03	1.0E-03	1.1E-03	9.5E-04	9.9E-04	9.8E-04	6.5E-05	1.0E-03	8.3E-05	1.030	0.654	
A_0056	*N*-Acetyl glucosamine 6-phosphate	C00357	HMDB01062	1.2E-04	9.6E-05	8.0E-05	1.3E-04	9.3E-05	7.4E-05	9.7E-05	1.8E-05	1.0E-04	3.0E-05	1.025	0.909	
A_0083	CDP-choline	C00307	HMDB01413	2.0E-04	1.8E-04	2.6E-04	2.2E-04	1.8E-04	2.4E-04	2.1E-04	4.0E-05	2.1E-04	3.1E-05	1.012	0.935	
A_0002	Propionic acid	C00163	HMDB00237	1.7E-04	9.9E-05	1.1E-04	1.4E-04	1.3E-04	1.0E-04	1.3E-04	4.0E-05	1.3E-04	2.1E-05	1.010	0.964	
A_0092	GDP-mannose GDP-galactose	C00096 C02280	HMDB01163 -	1.8E-04	2.2E-04	2.2E-04	2.1E-04	1.9E-04	2.4E-04	2.1E-04	2.3E-05	2.1E-04	2.4E-05	1.005	0.955
A_0017	Malic acid	C00149, C00497, C00711	HMDB00156, HMDB00744	1.5E-02	1.6E-02	1.5E-02	1.5E-02	1.3E-02	1.7E-02	1.5E-02	6.0E-04	1.5E-02	1.8E-03	1.001	0.992	
A_0010	Fumaric acid	C00122	HMDB00134	1.9E-03	2.1E-03	1.9E-03	1.8E-03	1.7E-03	2.4E-03	2.0E-03	1.0E-04	2.0E-03	3.5E-04	0.997	0.977	
A_0016	*N*-Acetyl-β-alanine	C01073	–	5.8E-05	7.2E-05	6.8E-05	6.8E-05	6.1E-05	6.6E-05	6.6E-05	7.3E-06	6.5E-05	3.7E-06	0.987	0.870	
A_0091	ADP-glucose GDP-fucose	C00498 C00325	HMDB06557 HMDB01095	8.2E-05	8.5E-05	8.9E-05	9.7E-05	7.9E-05	7.8E-05	8.6E-05	3.6E-06	8.5E-05	1.0E-05	0.987	0.873
A_0035	*N*-Acetyl glutamic acid	C00624	HMDB01138	7.1E-04	6.6E-04	6.4E-04	6.9E-04	6.6E-04	6.1E-04	6.7E-04	3.6E-05	6.5E-04	4.1E-05	0.981	0.714	
A_0029	*cis*-Aconitic acid	C00417	HMDB00072	1.6E-04	2.8E-04	1.5E-04	1.6E-04	1.9E-04	2.3E-04	2.0E-04	7.3E-05	1.9E-04	3.3E-05	0.974	0.920	
A_0043	Ribulose 5-phosphate	C00199, C01101	HMDB00618	2.5E-03	2.1E-03	1.7E-03	2.1E-03	2.3E-03	1.8E-03	2.1E-03	3.9E-04	2.1E-03	2.4E-04	0.969	0.817	
A_0068	CoA_divalent	C00010	HMDB01423	4.1E-04	4.1E-04	4.4E-04	3.8E-04	3.8E-04	4.1E-04	4.2E-04	2.0E-05	3.9E-04	2.0E-05	0.927	0.138	
A_0022	Pelargonic acid	C01601	HMDB00847	8.7E-05	5.5E-05	6.7E-05	5.3E-05	5.4E-05	8.7E-05	7.0E-05	1.6E-05	6.5E-05	1.9E-05	0.927	0.743	
A_0006	Lactic acid	C00186, C00256, C01432	HMDB00190, HMDB01311	2.9E-01	2.8E-01	3.1E-01	2.7E-01	2.5E-01	3.0E-01	3.0E-01	1.3E-02	2.7E-01	2.7E-02	0.920	0.262	
A_0094	CMP-*N*-acetylneuraminate	C00128	HMDB01176	4.7E-04	5.7E-04	5.2E-04	4.9E-04	5.4E-04	3.9E-04	5.2E-04	4.9E-05	4.7E-04	7.7E-05	0.919	0.481	
A_0031	Ascorbic acid	C00072	HMDB00044	3.5E-02	3.5E-02	3.2E-02	3.3E-02	2.8E-02	3.3E-02	3.4E-02	1.6E-03	3.1E-02	3.0E-03	0.918	0.255	
A_0061	cAMP	C00575	HMDB00058	2.7E-05	4.3E-05	3.5E-05	3.9E-05	2.3E-05	3.4E-05	3.5E-05	7.8E-06	3.2E-05	7.9E-06	0.916	0.667	
A_0085	ATP	C00002	HMDB00538	1.1E-03	1.1E-03	1.0E-03	1.1E-03	1.0E-03	7.9E-04	1.1E-03	3.4E-05	9.7E-04	1.7E-04	0.915	0.455	
A_0070	FAD_divalent	C00016	HMDB01248	5.9E-05	7.3E-05	7.6E-05	6.5E-05	6.3E-05	5.7E-05	6.9E-05	9.2E-06	6.2E-05	4.0E-06	0.890	0.288	
A_0090	UDP-glucuronic acid	C00167	HMDB00935	1.2E-04	1.4E-04	1.2E-04	1.1E-04	1.1E-04	1.2E-04	1.2E-04	1.3E-05	1.1E-04	4.9E-06	0.879	0.172	
A_0051	Glucose 1-phosphate	C00103	HMDB01586	1.5E-04	2.0E-04	2.0E-04	1.8E-04	1.3E-04	1.7E-04	1.8E-04	2.9E-05	1.6E-04	2.3E-05	0.869	0.328	
A_0018	Threonic acid	C01620	HMDB00943	1.2E-03	1.4E-03	1.3E-03	1.3E-03	1.0E-03	1.1E-03	1.3E-03	8.1E-05	1.1E-03	1.8E-04	0.860	0.211	
A_0057	*N*-Acetylneuraminic acid	C00270	HMDB00230	1.1E-03	1.0E-03	1.0E-03	9.2E-04	8.9E-04	8.1E-04	1.0E-03	4.3E-05	8.7E-04	5.6E-05	0.846	0.020	*
A_0014	Isethionic acid	C05123	HMDB03903	8.2E-04	9.5E-04	1.1E-03	7.7E-04	8.8E-04	7.6E-04	9.5E-04	1.3E-04	8.0E-04	6.6E-05	0.846	0.172	
A_0089	UDP-glucose UDP-galactose	C00029 C00052	HMDB00286 HMDB00302	1.2E-03	1.5E-03	1.3E-03	1.0E-03	1.1E-03	1.3E-03	1.3E-03	1.3E-04	1.1E-03	1.1E-04	0.846	0.110
A_0030	*N*-Acetylaspartic acid	C01042	HMDB00812	2.0E-01	2.0E-01	2.2E-01	1.8E-01	1.6E-01	1.7E-01	2.1E-01	1.0E-02	1.7E-01	8.5E-03	0.838	0.014	*
A_0073	UDP	C00015	HMDB00295	1.0E-04	1.2E-04	1.2E-04	1.1E-04	8.5E-05	9.2E-05	1.2E-04	1.1E-05	9.6E-05	1.4E-05	0.833	0.135	
A_0009	3-Hydroxybutyric acid	C01089, C03197	HMDB00011, HMDB00357, HMDB00442	9.8E-04	1.2E-03	8.5E-04	7.7E-04	8.8E-04	8.4E-04	1.0E-03	1.6E-04	8.3E-04	5.6E-05	0.833	0.196	
A_0054	Sedoheptulose 7-phosphate	C05382	HMDB01068	9.5E-05	1.1E-04	1.2E-04	6.6E-05	1.0E-04	1.1E-04	1.1E-04	1.4E-05	9.1E-05	2.2E-05	0.831	0.298	
A_0042	Pantothenic acid	C00864	HMDB00210	4.1E-04	4.5E-04	4.5E-04	3.2E-04	3.7E-04	3.8E-04	4.4E-04	2.1E-05	3.6E-04	3.2E-05	0.819	0.029	*
A_0038	Gluconic acid	C00257	HMDB00625	1.3E-04	1.1E-04	1.4E-04	1.1E-04	1.0E-04	9.8E-05	1.3E-04	1.3E-05	1.0E-04	6.6E-06	0.816	0.063	
A_0040	Lauric acid	C02679	HMDB00638	1.5E-04	1.2E-04	1.5E-04	1.5E-04	1.1E-04	8.5E-05	1.4E-04	1.6E-05	1.1E-04	3.2E-05	0.816	0.308	
A_0064	AMP	C00020	HMDB00045	1.6E-02	2.1E-02	2.3E-02	1.7E-02	1.3E-02	1.9E-02	2.0E-02	3.7E-03	1.6E-02	3.3E-03	0.810	0.248	
A_0046	Biotin	C00120	HMDB00030	2.1E-04	2.4E-04	2.2E-04	1.7E-04	2.0E-04	1.6E-04	2.2E-04	1.7E-05	1.8E-04	1.8E-05	0.804	0.038	*
A_0055	*N*-Acetylglucosamine 1-phosphate	C04256	HMDB01367	1.3E-04	1.2E-04	1.2E-04	1.2E-04	9.2E-05	9.0E-05	1.3E-04	6.0E-06	1.0E-04	1.9E-05	0.803	0.147	
A_0050	*myo*-Inositol 2-phosphate	–	–	7.5E-04	8.0E-04	9.6E-04	5.4E-04	6.3E-04	8.3E-04	8.4E-04	1.1E-04	6.7E-04	1.5E-04	0.799	0.191	
A_0045	2-Deoxyglucose 6-phosphate	C06369	–	8.4E-05	1.3E-04	6.9E-05	9.6E-05	5.2E-05	7.6E-05	9.4E-05	3.1E-05	7.4E-05	2.2E-05	0.793	0.434	
A_0005	Butyric acid	C00246	HMDB00039	3.9E-05	5.5E-05	7.0E-05	4.6E-05	5.0E-05	3.4E-05	5.5E-05	1.5E-05	4.3E-05	8.2E-06	0.789	0.333	
A_0059	CMP	C00055	HMDB00095	2.1E-04	3.5E-04	3.3E-04	2.1E-04	1.7E-04	3.0E-04	3.0E-04	7.6E-05	2.3E-04	6.8E-05	0.757	0.286	
A_0076	ADP	C00008	HMDB01341	4.7E-03	5.7E-03	4.9E-03	3.8E-03	3.7E-03	4.0E-03	5.1E-03	5.4E-04	3.8E-03	1.3E-04	0.755	0.050	*
A_0078	Adenylosuccinic acid	C03794	HMDB00536	6.4E-04	8.0E-04	7.4E-04	4.6E-04	4.7E-04	7.1E-04	7.2E-04	8.1E-05	5.4E-04	1.4E-04	0.753	0.153	
A_0019	Ethanolamine phosphate	C00346	HMDB00224	1.3E-02	1.6E-02	1.8E-02	1.2E-02	1.1E-02	1.2E-02	1.6E-02	2.3E-03	1.2E-02	3.9E-04	0.743	0.091	
A_0066	GMP	C00144	HMDB01397	2.5E-03	3.3E-03	3.3E-03	2.1E-03	2.0E-03	2.5E-03	3.0E-03	4.8E-04	2.2E-03	2.6E-04	0.730	0.079	
A_0012	Succinic acid	C00042	HMDB00254	2.0E-02	2.1E-02	1.9E-02	1.5E-02	1.6E-02	1.3E-02	2.0E-02	1.3E-03	1.4E-02	1.7E-03	0.719	0.012	*
A_0021	3-Phenylpropionic acid	C05629	HMDB00764	1.3E-04	1.3E-04	1.1E-04	8.6E-05	8.0E-05	9.8E-05	1.2E-04	1.3E-05	8.8E-05	9.0E-06	0.715	0.023	*
A_0095	NAD^+^	C00003	HMDB00902	1.3E-03	1.9E-03	1.7E-03	1.2E-03	9.6E-04	1.2E-03	1.6E-03	3.2E-04	1.1E-03	1.5E-04	0.702	0.107	
A_0027	Dihydroxyacetone phosphate	C00111	HMDB01473	1.9E-04	2.0E-04	2.2E-04	1.2E-04	1.7E-04	1.3E-04	2.0E-04	1.2E-05	1.4E-04	2.3E-05	0.694	0.024	*
A_0065	IMP	C00130	HMDB00175	2.5E-03	2.8E-03	2.7E-03	2.1E-03	1.7E-03	1.7E-03	2.7E-03	1.7E-04	1.8E-03	2.6E-04	0.691	0.015	*
A_0008	2-Hydroxybutyric acid	C05984	HMDB00008	7.5E-05	8.4E-05	9.3E-05	5.1E-05	6.7E-05	5.6E-05	8.4E-05	8.9E-06	5.8E-05	8.3E-06	0.688	0.021	*
A_0097	NADP^+^	C00006	HMDB00217	8.3E-05	9.0E-05	8.9E-05	7.0E-05	4.4E-05	5.7E-05	8.7E-05	3.8E-06	5.7E-05	1.3E-05	0.654	0.045	*
A_0096	NADH	C00004	HMDB01487	1.9E-04	2.1E-04	2.3E-04	1.5E-04	1.5E-04	1.1E-04	2.1E-04	2.3E-05	1.3E-04	2.5E-05	0.646	0.020	*
A_0060	UMP	C00105	HMDB00288	5.4E-04	1.1E-03	1.1E-03	5.4E-04	4.6E-04	7.5E-04	9.1E-04	3.3E-04	5.8E-04	1.5E-04	0.641	0.220	
A_0034	3-Phosphoglyceric acid	C00197	HMDB00807	6.6E-04	5.3E-04	4.7E-04	3.3E-04	3.7E-04	3.3E-04	5.5E-04	9.5E-05	3.4E-04	2.3E-05	0.620	0.055	
A_0028	Glycerol 3-phosphate	C00093	HMDB00126	4.0E-03	3.4E-03	3.9E-03	2.4E-03	3.5E-03	1.0E-03	3.8E-03	2.9E-04	2.3E-03	1.3E-03	0.612	0.178	
A_0047	Glucose 6-phosphate	C00668, C01172, C00092	HMDB01401	8.0E-05	1.0E-04	1.2E-04	5.8E-05	5.9E-05	6.4E-05	1.0E-04	2.0E-05	6.0E-05	2.9E-06	0.598	0.072	
A_0049	Fructose 6-phosphate	C00085	HMDB00124	2.8E-05	1.8E-05	3.4E-05	1.7E-05	1.6E-05	1.3E-05	2.7E-05	7.9E-06	1.5E-05	2.0E-06	0.574	0.122	
A_0062	Fructose 1,6-diphosphate	C00354	HMDB01058	4.7E-04	4.4E-04	7.2E-04	2.7E-04	3.0E-04	2.2E-04	5.4E-04	1.5E-04	2.7E-04	4.1E-05	0.491	0.079	
A_0086	Taurocholic acid	C05122	HMDB00036	1.2E-04	1.7E-04	1.6E-04	3.1E-05	1.4E-05	ND	1.5E-04	3.1E-05	2.3E-05	1.2E-05	0.150	0.010	**

*^1^Ex-GF/GF ratio*.

*^2^Welch’s *t*-test (**p* < 0.05, ***p* < 0.01)*.

*ND, not detected*.

**Table A2:** **Cationic metabolites detected from cerebrum in GF mice and Ex-GF mice**.

ID	HMT DB^†^			Relative area of standard										Comparative analysis		
	Compound name	KEGG ID	HMDB ID	GF			Ex-GF			GF		Ex-GF				
				GF1	GF2	GF3	Ex-GF1	Ex-GF2	Ex-GF3	Mean	SD	Mean	SD	Ratio^1^	*p*-Value^2^	
C_0003	Trimethylamine *N*-oxide	C01104	HMDB00925	2.2E-05	1.6E-05	1.8E-05	8.9E-05	9.3E-05	6.4E-05	1.9E-05	3.4E-06	8.2E-05	1.5E-05	4.390	0.015	*
C_0074	*N*^5^-Ethylglutamine	C01047	–	6.8E-05	5.5E-05	5.9E-05	1.6E-04	1.2E-04	1.5E-04	6.1E-05	6.6E-06	1.4E-04	2.4E-05	2.361	0.022	*
C_0123	Cysteine glutathione disulfide	–	HMDB00656	6.3E-04	2.1E-04	9.6E-05	8.1E-04	1.0E-03	1.9E-04	3.1E-04	2.8E-04	6.8E-04	4.4E-04	2.173	0.299	
C_0031	Cys	C00097, C00736, C00793	HMDB00574, HMDB03417	1.7E-03	5.6E-04	3.6E-04	2.6E-03	1.3E-03	7.3E-04	8.6E-04	7.1E-04	1.5E-03	9.5E-04	1.790	0.381	
C_0029	2-Methylserine	C02115	–	4.4E-05	5.1E-05	5.2E-05	1.0E-04	7.7E-05	8.3E-05	4.9E-05	4.2E-06	8.7E-05	1.2E-05	1.775	0.025	*
C_0073	3-Methylhistidine	C01152	HMDB00479	5.4E-04	6.6E-04	6.5E-04	1.2E-03	9.5E-04	9.5E-04	6.1E-04	6.7E-05	1.0E-03	1.3E-04	1.677	0.018	*
C_0099	Cystine	C00491, C01420	HMDB00192	2.0E-05	ND	ND	2.9E-05	3.7E-05	ND	2.0E-05	NA	3.3E-05	5.5E-06	1.673	NA	
C_0018	Hypotaurine	C00519	HMDB00965	6.5E-04	4.2E-04	4.9E-04	9.5E-04	6.9E-04	7.4E-04	5.2E-04	1.2E-04	7.9E-04	1.4E-04	1.524	0.059	
C_0089	Trp	C00078, C00525, C00806	HMDB00929	1.0E-03	9.8E-04	9.4E-04	1.3E-03	1.5E-03	1.5E-03	9.7E-04	2.7E-05	1.4E-03	1.1E-04	1.480	0.015	*
C_0035	Pipecolic acid	C00408	HMDB00070, HMDB00716, HMDB05960	1.6E-04	1.6E-04	1.6E-04	2.2E-04	2.5E-04	2.3E-04	1.6E-04	7.4E-07	2.3E-04	1.3E-05	1.444	0.010	*
C_0044	Thiaproline	–	–	1.0E-03	5.9E-04	2.8E-04	1.1E-03	9.6E-04	5.4E-04	6.2E-04	3.6E-04	8.7E-04	3.0E-04	1.405	0.410	
C_0032	Nicotinamide	C00153	HMDB01406	7.3E-03	4.6E-03	5.5E-03	7.7E-03	8.9E-03	7.6E-03	5.8E-03	1.4E-03	8.0E-03	7.4E-04	1.385	0.093	
C_0109	Inosine	C00294	HMDB00195	1.5E-02	8.3E-03	6.6E-03	1.6E-02	1.7E-02	8.4E-03	1.0E-02	4.5E-03	1.4E-02	4.7E-03	1.378	0.371	
C_0079	Tyr	C00082, C01536, C06420	HMDB00158	3.3E-03	3.3E-03	3.8E-03	4.3E-03	5.0E-03	4.9E-03	3.5E-03	3.2E-04	4.8E-03	3.8E-04	1.371	0.012	*
C_0060	*Threo*-β-Methyl aspartic acid	C03618	-	9.4E-05	6.7E-05	6.6E-05	9.5E-05	1.1E-04	1.0E-04	7.6E-05	1.6E-05	1.0E-04	6.5E-06	1.339	0.091	
C_0072	Phe	C00079, C02057, C02265	HMDB00159	3.6E-03	3.9E-03	3.7E-03	4.9E-03	5.1E-03	4.9E-03	3.7E-03	1.4E-04	5.0E-03	1.4E-04	1.330	0.000	***
C_0053	1-Methyl-4-imidazoleacetic acid	C05828	HMDB02820	9.5E-05	7.5E-05	1.0E-04	1.1E-04	1.5E-04	9.5E-05	9.1E-05	1.5E-05	1.2E-04	2.8E-05	1.319	0.209	
C_0047	Hypoxanthine	C00262	HMDB00157	8.9E-03	5.6E-03	4.2E-03	9.7E-03	9.4E-03	5.0E-03	6.2E-03	2.4E-03	8.0E-03	2.6E-03	1.292	0.431	
C_0065	Guanine	C00242	HMDB00132	2.1E-05	2.1E-05	1.9E-05	2.2E-05	3.1E-05	2.3E-05	2.0E-05	1.4E-06	2.6E-05	5.0E-06	1.263	0.202	
C_0045	Asp	C00049, C00402, C16433	HMDB00191, HMDB06483	2.7E-03	2.7E-03	2.7E-03	3.6E-03	3.4E-03	3.4E-03	2.7E-03	2.8E-05	3.4E-03	1.2E-04	1.259	0.007	**
C_0112	Guanosine	C00387	HMDB00133	1.8E-03	1.1E-03	1.0E-03	1.8E-03	1.9E-03	1.2E-03	1.3E-03	4.1E-04	1.6E-03	3.9E-04	1.233	0.405	
C_0086	SDMA	–	HMDB03334	2.4E-05	2.4E-05	2.0E-05	2.8E-05	3.1E-05	2.4E-05	2.3E-05	2.2E-06	2.8E-05	3.7E-06	1.215	0.139	
C_0058	Spermidine	C00315	HMDB01257	9.9E-04	9.4E-04	1.1E-03	1.0E-03	1.5E-03	1.2E-03	1.0E-03	1.0E-04	1.2E-03	2.6E-04	1.200	0.308	
C_0116	Arg-Glu	–	–	8.2E-06	7.6E-06	8.3E-06	8.3E-06	9.5E-06	1.1E-05	8.0E-06	3.5E-07	9.6E-06	1.3E-06	1.194	0.167	
C_0061	Gln	C00064, C00303, C00819	HMDB00641, HMDB03423	7.0E-03	7.0E-03	7.3E-03	8.2E-03	8.2E-03	9.0E-03	7.1E-03	1.7E-04	8.5E-03	4.6E-04	1.188	0.025	*
C_0008	1-Methyl-2-pyrrolidone	C11118	–	1.2E-04	9.6E-05	1.0E-04	1.2E-04	1.1E-04	1.4E-04	1.1E-04	1.1E-05	1.2E-04	1.5E-05	1.170	0.171	
C_0019	Cytosine	C00380	HMDB00630	4.1E-06	3.1E-06	3.3E-06	4.2E-06	4.1E-06	3.9E-06	3.5E-06	5.4E-07	4.1E-06	1.8E-07	1.152	0.220	
C_0013	2-Aminoisobutyric acid	C03665	HMDB01906	9.8E-05	1.2E-04	9.4E-05	1.3E-04	1.1E-04	1.2E-04	1.0E-04	1.3E-05	1.2E-04	7.3E-06	1.141	0.177	
C_0094	Carnosine	C00386	HMDB00033	2.4E-03	2.4E-03	2.3E-03	3.0E-03	2.9E-03	2.2E-03	2.4E-03	7.3E-05	2.7E-03	4.6E-04	1.140	0.340	
C_0021	Uracil	C00106	HMDB00300	3.3E-04	2.9E-04	2.6E-04	3.8E-04	3.6E-04	2.6E-04	2.9E-04	3.9E-05	3.3E-04	6.0E-05	1.139	0.392	
C_0093	Cystathionine	C00542, C02291	HMDB00099	6.6E-04	4.4E-04	6.6E-04	5.7E-04	8.3E-04	6.0E-04	5.9E-04	1.2E-04	6.7E-04	1.4E-04	1.137	0.497	
C_0067	His	C00135, C00768, C06419	HMDB00177	5.5E-03	5.9E-03	5.4E-03	6.1E-03	6.1E-03	6.8E-03	5.6E-03	2.5E-04	6.3E-03	4.3E-04	1.135	0.071	
C_0100	Homocarnosine	C00884	HMDB00745	5.1E-03	4.7E-03	5.5E-03	5.4E-03	6.4E-03	5.6E-03	5.1E-03	4.3E-04	5.8E-03	5.4E-04	1.130	0.173	
C_0014	Choline	C00114	HMDB00097	2.3E-02	2.0E-02	1.4E-02	2.7E-02	2.3E-02	1.4E-02	1.9E-02	4.6E-03	2.1E-02	6.3E-03	1.118	0.643	
C_0020	Histamine	C00388	HMDB00870	2.5E-05	2.5E-05	3.0E-05	2.3E-05	4.1E-05	2.6E-05	2.7E-05	3.2E-06	3.0E-05	9.3E-06	1.116	0.630	
C_0046	Adenine	C00147	HMDB00034	3.6E-04	2.9E-04	2.5E-04	3.2E-04	3.9E-04	2.9E-04	3.0E-04	5.4E-05	3.3E-04	5.1E-05	1.114	0.470	
C_0122	*S*-Adenosyl methionine	C00019	HMDB01185	4.9E-04	4.4E-04	5.2E-04	5.2E-04	5.5E-04	5.4E-04	4.8E-04	4.1E-05	5.4E-04	1.8E-05	1.114	0.134	
C_0030	Betaine aldehyde + H_2_O	C00576	HMDB01252	1.7E-05	1.4E-05	1.2E-05	1.9E-05	1.5E-05	1.3E-05	1.5E-05	2.6E-06	1.6E-05	3.0E-06	1.108	0.532	
C_0034	1-Methylhistamine	C05127	HMDB00898	7.1E-05	4.2E-05	6.7E-05	5.6E-05	7.7E-05	6.6E-05	6.0E-05	1.6E-05	6.6E-05	1.1E-05	1.104	0.610	
C_0114	Argininosuccinic acid	C03406	HMDB00052	2.0E-04	1.9E-04	1.8E-04	1.9E-04	2.1E-04	2.2E-04	1.9E-04	9.3E-06	2.1E-04	1.7E-05	1.099	0.191	
C_0078	Serotonin	C00780	HMDB00259	4.4E-05	4.4E-05	4.4E-05	3.6E-05	6.4E-05	4.5E-05	4.4E-05	3.1E-07	4.8E-05	1.4E-05	1.099	0.649	
C_0103	Uridine	C00299	HMDB00296	1.5E-03	1.2E-03	1.2E-03	1.4E-03	1.6E-03	1.3E-03	1.3E-03	1.8E-04	1.4E-03	1.8E-04	1.096	0.443	
C_0087	Spermine	C00750	HMDB01256	7.6E-05	9.7E-05	7.7E-05	8.6E-05	7.9E-05	1.1E-04	8.3E-05	1.2E-05	9.1E-05	1.4E-05	1.091	0.524	
C_0002	Gly	C00037	HMDB00123	2.1E-02	1.7E-02	1.6E-02	1.9E-02	2.4E-02	1.7E-02	1.8E-02	2.3E-03	2.0E-02	3.7E-03	1.090	0.563	
C_0076	Arg	C00062, C00792	HMDB00517, HMDB03416	8.6E-03	6.7E-03	8.8E-03	8.3E-03	9.4E-03	8.5E-03	8.0E-03	1.2E-03	8.7E-03	6.2E-04	1.087	0.429	
C_0023	Pro	C00148, C00763, C16435	HMDB00162, HMDB03411	6.6E-03	6.2E-03	6.3E-03	7.5E-03	6.7E-03	6.4E-03	6.4E-03	2.3E-04	6.9E-03	5.8E-04	1.076	0.280	
C_0063	Met	C00073, C00855, C01733	HMDB00696	1.9E-03	1.7E-03	2.2E-03	2.1E-03	2.0E-03	2.1E-03	1.9E-03	2.2E-04	2.1E-03	4.5E-05	1.075	0.375	
C_0113	His-Glu	–	–	3.9E-06	3.0E-06	N.D.	3.7E-06	3.5E-06	4.0E-06	3.5E-06	6.4E-07	3.7E-06	2.8E-07	1.068	0.695	
C_0071	Methionine sulfoxide	C02989	HMDB02005	2.5E-04	1.8E-04	2.7E-04	2.1E-04	2.9E-04	2.5E-04	2.3E-04	4.7E-05	2.5E-04	4.2E-05	1.067	0.691	
C_0108	Adenosine	C00212	HMDB00050	2.3E-02	2.2E-02	2.3E-02	2.5E-02	2.3E-02	2.4E-02	2.2E-02	9.2E-04	2.4E-02	5.6E-04	1.062	0.102	
C_0005	β-Ala	C00099	HMDB00056	2.2E-03	1.9E-03	2.1E-03	1.8E-03	2.6E-03	2.1E-03	2.1E-03	1.8E-04	2.2E-03	3.9E-04	1.062	0.650	
C_0105	γ-Glu-Cys	C00669	HMDB01049	1.3E-04	1.1E-04	1.3E-04	1.6E-04	1.2E-04	1.2E-04	1.2E-04	1.5E-05	1.3E-04	2.1E-05	1.060	0.650	
C_0110	Saccharopine	C00449	HMDB00279	3.8E-04	3.5E-04	3.7E-04	2.9E-04	4.9E-04	3.7E-04	3.7E-04	1.7E-05	3.9E-04	1.0E-04	1.058	0.754	
C_0082	*N*^6^-Acetyllysine	C02727	HMDB00206	2.8E-05	2.3E-05	3.0E-05	2.9E-05	3.0E-05	2.7E-05	2.7E-05	3.9E-06	2.9E-05	1.8E-06	1.053	0.605	
C_0015	GABA	C00334	HMDB00112	5.7E-03	4.6E-03	4.9E-03	4.4E-03	6.2E-03	5.3E-03	5.1E-03	5.9E-04	5.3E-03	9.1E-04	1.048	0.722	
C_0068	2-Aminoadipic acid	C00956	HMDB00510	1.5E-03	1.9E-03	2.0E-03	1.8E-03	1.7E-03	2.2E-03	1.8E-03	2.7E-04	1.9E-03	2.9E-04	1.048	0.726	
C_0040	Asn	C00152, C01905, C16438	HMDB00168	3.4E-03	3.5E-03	3.7E-03	3.8E-03	3.6E-03	3.6E-03	3.5E-03	1.7E-04	3.7E-03	1.3E-04	1.046	0.275	
C_0097	Thr-Asp	–	–	1.2E-05	1.4E-05	1.1E-05	1.1E-05	1.4E-05	1.4E-05	1.2E-05	1.5E-06	1.3E-05	2.0E-06	1.045	0.721	
C_0075	*N*-Acetylornithine	C00437	HMDB03357	1.3E-05	8.3E-06	ND	1.1E-05	ND	ND	1.1E-05	3.5E-06	1.1E-05	NA	1.043	NA	
C_0025	Betaine	C00719	HMDB00043	9.6E-04	8.3E-04	9.5E-04	9.2E-04	1.1E-03	8.3E-04	9.1E-04	7.0E-05	9.5E-04	1.4E-04	1.042	0.696	
C_0010	Homoserine lactone	–	–	7.8E-05	6.8E-05	8.0E-05	7.3E-05	7.7E-05	8.4E-05	7.5E-05	6.9E-06	7.8E-05	5.6E-06	1.032	0.660	
C_0107	Thiamine	C00378	HMDB00235	6.8E-05	6.0E-05	5.3E-05	6.7E-05	6.2E-05	5.8E-05	6.0E-05	7.7E-06	6.2E-05	4.7E-06	1.029	0.753	
C_0111	1-Methyladenosine	C02494	HMDB03331	9.6E-05	1.1E-04	1.5E-04	1.2E-04	1.2E-04	1.2E-04	1.2E-04	2.6E-05	1.2E-04	3.8E-06	1.028	0.850	
C_0064	Triethanolamine	C06771	–	1.1E-05	1.2E-05	1.4E-05	1.5E-05	1.2E-05	1.2E-05	1.2E-05	1.5E-06	1.3E-05	1.6E-06	1.028	0.799	
C_0026	Val	C00183, C06417, C16436	HMDB00883	8.5E-03	7.5E-03	7.5E-03	7.8E-03	8.2E-03	8.0E-03	7.8E-03	5.5E-04	8.0E-03	1.9E-04	1.022	0.653	
C_0056	Acetylcholine	C01996	HMDB00895	4.5E-04	5.0E-04	6.0E-04	4.8E-04	4.9E-04	6.1E-04	5.2E-04	7.8E-05	5.3E-04	7.3E-05	1.021	0.869	
C_0115	5′-Deoxy-5′-methyl thioadenosine	C00170	HMDB01173	1.9E-05	1.6E-05	1.7E-05	2.1E-05	1.6E-05	1.6E-05	1.8E-05	1.3E-06	1.8E-05	2.9E-06	1.016	0.891	
C_0054	Stachydrine	C10172	HMDB04827	1.3E-04	9.7E-05	1.4E-04	1.5E-04	1.3E-04	8.6E-05	1.2E-04	2.2E-05	1.2E-04	3.4E-05	1.014	0.946	
C_0085	Gly-Asp	-	-	8.2E-05	9.0E-05	8.5E-05	1.0E-04	8.4E-05	7.3E-05	8.6E-05	4.3E-06	8.6E-05	1.4E-05	1.009	0.935	
C_0039	Leu	C00123, C01570, C16439	HMDB00687	9.5E-03	8.3E-03	8.4E-03	8.9E-03	9.0E-03	8.4E-03	8.7E-03	7.0E-04	8.8E-03	3.1E-04	1.004	0.941	
C_0080	Phosphorylcholine	C00588	HMDB01565	1.6E-02	1.6E-02	1.8E-02	1.6E-02	1.6E-02	1.6E-02	1.6E-02	1.1E-03	1.6E-02	2.3E-04	0.994	0.891	
C_0036	*trans*-Glutaconic acid	C02214	HMDB00620	9.0E-05	9.7E-05	9.9E-05	7.7E-05	1.1E-04	9.7E-05	9.5E-05	4.7E-06	9.4E-05	1.7E-05	0.992	0.945	
C_0090	Carboxymethyl lysine	–	–	5.5E-05	5.2E-05	5.2E-05	5.1E-05	5.7E-05	5.0E-05	5.3E-05	2.1E-06	5.2E-05	3.7E-06	0.989	0.818	
C_0095	2′-Deoxycytidine	C00881	HMDB00014	3.3E-05	2.2E-05	2.6E-05	3.5E-05	2.7E-05	1.9E-05	2.7E-05	5.6E-06	2.7E-05	7.8E-06	0.988	0.955	
C_0038	Ile	C00407, C06418, C16434	HMDB00172	4.8E-03	4.2E-03	4.1E-03	4.4E-03	4.3E-03	4.3E-03	4.4E-03	4.1E-04	4.3E-03	6.2E-05	0.987	0.841	
C_0012	2-Aminobutyric acid	C02261, C02356	HMDB00452	1.7E-04	1.4E-04	1.7E-04	1.7E-04	1.5E-04	1.6E-04	1.6E-04	2.0E-05	1.6E-04	7.1E-06	0.987	0.879	
C_0062	Glu	C00025, C00217, C00302	HMDB00148, HMDB03339	1.4E-02	1.4E-02	1.5E-02	1.4E-02	1.3E-02	1.5E-02	1.4E-02	4.6E-04	1.4E-02	6.3E-04	0.987	0.698	
C_0022	Creatinine	C00791	HMDB00562	6.6E-04	5.9E-04	6.5E-04	5.6E-04	6.3E-04	6.7E-04	6.3E-04	4.0E-05	6.2E-04	5.5E-05	0.978	0.745	
C_0083	Gly-Leu	C02155	HMDB00759	1.5E-04	1.4E-04	1.5E-04	1.6E-04	1.4E-04	1.3E-04	1.5E-04	9.7E-06	1.4E-04	1.4E-05	0.978	0.759	
C_0059	Lys	C00047, C00739, C16440	HMDB00182, HMDB03405	1.1E-02	9.5E-03	1.0E-02	9.8E-03	1.1E-02	1.0E-02	1.0E-02	8.8E-04	1.0E-02	4.4E-04	0.977	0.702	
C_0084	*N*^6^,*N*^6^,*N*^6^-Trimethyllysine	C03793	HMDB01325	3.4E-04	4.3E-04	4.1E-04	4.0E-04	3.3E-04	4.2E-04	3.9E-04	4.7E-05	3.8E-04	4.7E-05	0.977	0.823	
C_0104	Pyridoxamine 5′-phosphate	C00647	HMDB01555	3.4E-04	3.5E-04	3.7E-04	3.2E-04	3.7E-04	3.5E-04	3.5E-04	1.4E-05	3.5E-04	2.4E-05	0.976	0.641	
C_0117	Glutathione (GSSG)_divalent	C00127	HMDB03337	1.8E-02	2.0E-02	1.7E-02	1.7E-02	2.1E-02	1.6E-02	1.9E-02	1.4E-03	1.8E-02	2.7E-03	0.973	0.794	
C_0092	*N*-Acetylglucosamine	C00140	HMDB00215	1.5E-04	1.6E-04	1.4E-04	1.6E-04	1.5E-04	1.3E-04	1.5E-04	6.8E-06	1.5E-04	1.3E-05	0.971	0.639	
C_0069	Carnitine	C00318, C00487, C15025	HMDB00062	8.6E-03	7.7E-03	7.9E-03	7.4E-03	9.0E-03	7.1E-03	8.1E-03	4.9E-04	7.8E-03	1.0E-03	0.969	0.736	
C_0001	Urea	C00086	HMDB00294	7.5E-02	7.4E-02	7.7E-02	7.0E-02	7.3E-02	7.5E-02	7.5E-02	1.6E-03	7.3E-02	2.7E-03	0.968	0.274	
C_0042	Creatine	C00300	HMDB00064	2.2E-02	2.2E-02	2.3E-02	2.1E-02	2.1E-02	2.2E-02	2.2E-02	4.4E-04	2.1E-02	3.2E-04	0.965	0.078	
C_0051	Tyramine	C00483	HMDB00306	1.2E-05	1.0E-05	2.0E-05	1.1E-05	1.5E-05	1.3E-05	1.4E-05	5.0E-06	1.3E-05	2.0E-06	0.962	0.878	
C_0007	Ala	C00041, C00133, C01401	HMDB00161, HMDB01310	1.1E-03	1.2E-03	1.2E-03	1.3E-03	9.6E-04	1.2E-03	1.2E-03	4.3E-05	1.1E-03	1.6E-04	0.960	0.667	
C_0077	Citrulline	C00327	HMDB00904	1.1E-03	1.0E-03	1.1E-03	1.2E-03	8.2E-04	1.1E-03	1.1E-03	6.9E-05	1.0E-03	1.9E-04	0.959	0.735	
C_0004	Putrescine	C00134	HMDB01414	1.2E-04	9.7E-05	1.0E-04	9.5E-05	1.0E-04	1.0E-04	1.1E-04	1.2E-05	1.0E-04	4.7E-06	0.951	0.544	
C_0106	Glycero phosphocholine	C00670	HMDB00086	1.8E-02	1.9E-02	2.1E-02	1.3E-02	2.1E-02	2.0E-02	1.9E-02	1.5E-03	1.8E-02	4.2E-03	0.947	0.725	
C_0027	Homoserine	C00263	HMDB00719	2.3E-04	2.6E-04	2.7E-04	2.6E-04	2.2E-04	2.4E-04	2.5E-04	2.0E-05	2.4E-04	1.8E-05	0.940	0.379	
C_0055	4-Guanidinobutyric acid	C01035	HMDB03464	3.3E-04	2.6E-04	3.3E-04	3.0E-04	3.0E-04	2.5E-04	3.1E-04	4.4E-05	2.8E-04	3.3E-05	0.929	0.534	
C_0119	Thiamine phosphate	C01081	HMDB02666	5.2E-05	4.8E-05	4.1E-05	4.3E-05	4.8E-05	3.9E-05	4.7E-05	5.6E-06	4.4E-05	4.4E-06	0.924	0.436	
C_0043	Ornithine	C00077, C00515, C01602	HMDB00214, HMDB03374	3.4E-04	2.7E-04	4.3E-04	3.5E-04	2.8E-04	3.2E-04	3.5E-04	7.7E-05	3.2E-04	3.5E-05	0.917	0.597	
C_0102	Cytidine	C00475	HMDB00089	2.0E-03	1.8E-03	1.6E-03	2.1E-03	1.4E-03	1.4E-03	1.8E-03	2.2E-04	1.6E-03	4.1E-04	0.891	0.520	
C_0033	Taurine	C00245	HMDB00251	2.8E-02	3.0E-02	3.2E-02	2.8E-02	2.4E-02	2.7E-02	3.0E-02	1.8E-03	2.7E-02	2.5E-03	0.885	0.128	
C_0028	Thr	C00188, C00820	HMDB00167	1.8E-02	1.8E-02	1.9E-02	1.4E-02	1.6E-02	1.7E-02	1.8E-02	4.3E-04	1.6E-02	1.3E-03	0.884	0.101	
C_0118	Glutathione (GSH)	C00051	HMDB00125	5.6E-03	6.1E-03	7.3E-03	5.5E-03	4.2E-03	6.7E-03	6.3E-03	8.6E-04	5.5E-03	1.2E-03	0.874	0.422	
C_0121	*S*-Adenosylhomo cysteine	C00021	HMDB00939	6.8E-05	7.6E-05	7.7E-05	6.7E-05	6.7E-05	5.9E-05	7.4E-05	5.0E-06	6.4E-05	4.8E-06	0.874	0.080	
C_0091	β-Ala-Lys	C05341	–	3.4E-05	2.4E-05	3.8E-05	2.4E-05	3.6E-05	2.4E-05	3.2E-05	7.1E-06	2.8E-05	7.0E-06	0.873	0.521	
C_0024	Guanidoacetic acid	C00581	HMDB00128	4.6E-04	3.8E-04	4.2E-04	4.0E-04	3.4E-04	3.3E-04	4.2E-04	4.1E-05	3.6E-04	3.8E-05	0.855	0.131	
C_0057	γ-Butyrobetaine	C01181	HMDB01161	1.8E-03	1.7E-03	1.8E-03	1.6E-03	1.3E-03	1.5E-03	1.8E-03	3.1E-05	1.5E-03	1.3E-04	0.841	0.059	
C_0017	*N*-Methylaniline	C02299	–	ND	1.6E-05	1.5E-05	1.1E-05	1.4E-05	ND	1.5E-05	9.7E-07	1.3E-05	2.2E-06	0.834	0.325	
C_0070	5-Hydroxylysine	C16741	HMDB00450	1.4E-05	1.0E-05	1.2E-05	1.1E-05	7.4E-06	1.1E-05	1.2E-05	1.8E-06	1.0E-05	2.3E-06	0.831	0.295	
C_0096	γ-Glu-2-aminobutyric acid	–	–	1.7E-04	1.6E-04	2.0E-04	1.5E-04	1.6E-04	1.3E-04	1.8E-04	1.9E-05	1.5E-04	1.3E-05	0.829	0.090	
C_0088	*O*-Acetylcarnitine	C02571	HMDB00201	5.0E-04	2.9E-04	4.6E-04	3.8E-04	3.3E-04	3.1E-04	4.2E-04	1.1E-04	3.4E-04	3.4E-05	0.816	0.370	
C_0041	Gly-Gly	C02037	HMDB11733	1.2E-04	1.4E-04	1.5E-04	1.1E-04	1.0E-04	1.2E-04	1.4E-04	1.3E-05	1.1E-04	1.1E-05	0.804	0.053	
C_0101	5,6,7,8-Tetrahydrobiopterin	C00272	HMDB00027	N.D.	1.3E-05	1.5E-05	7.9E-06	1.2E-05	1.4E-05	1.4E-05	1.6E-06	1.1E-05	3.1E-06	0.794	0.264	
C_0016	Ser	C00065, C00716, C00740	HMDB00187, HMDB03406	9.8E-04	1.1E-03	1.1E-03	8.2E-04	7.3E-04	8.3E-04	1.0E-03	6.5E-05	7.9E-04	5.5E-05	0.759	0.007	**
C_0081	*N*^8^-Acetylspermidine	C01029	HMDB02189	5.6E-05	4.6E-05	4.6E-05	3.9E-05	3.5E-05	3.6E-05	4.9E-05	6.0E-06	3.7E-05	2.0E-06	0.746	0.056	
C_0050	Trigonelline	C01004	HMDB00875	1.4E-04	9.8E-05	1.2E-04	1.0E-04	8.2E-05	7.4E-05	1.2E-04	1.8E-05	8.7E-05	1.6E-05	0.738	0.095	
C_0048	1-Methylnicotinamide	C02918	HMDB00699	4.5E-05	5.3E-05	5.4E-05	4.3E-05	3.4E-05	3.4E-05	5.0E-05	5.2E-06	3.7E-05	5.0E-06	0.736	0.033	*
C_0098	Ser-Glu	–	–	5.0E-05	4.1E-05	5.0E-05	3.5E-05	3.8E-05	2.9E-05	4.7E-05	5.5E-06	3.4E-05	4.8E-06	0.729	0.040	*
C_0052	Urocanic acid	C00785	HMDB00301	1.0E-04	3.6E-05	4.4E-05	4.1E-05	6.9E-05	1.2E-05	6.0E-05	3.5E-05	4.1E-05	2.8E-05	0.677	0.503	
C_0037	Hydroxyproline	C01015, C01157	HMDB06055, HMDB00725	1.6E-03	1.7E-03	1.6E-03	1.2E-03	9.9E-04	1.0E-03	1.6E-03	3.2E-05	1.1E-03	1.1E-04	0.651	0.009	**
C_0009	Cyclohexylamine	C00571	–	1.5E-05	1.9E-05	2.3E-05	1.4E-05	8.5E-06	1.4E-05	1.9E-05	4.1E-06	1.2E-05	3.4E-06	0.647	0.094	
C_0120	*S*-Lactoylglutathione	C03451	HMDB01066	ND	ND	1.8E-05	ND	ND	1.0E-05	1.8E-05	NA	1.0E-05	NA	0.584	NA	
C_0066	Dopamine	C03758	HMDB00073	2.9E-04	5.8E-04	7.1E-04	2.4E-04	2.7E-04	3.5E-04	5.3E-04	2.2E-04	2.9E-04	5.6E-05	0.543	0.188	

*^1^Ex-GF/GF ratio*.

*^2^Welch’s *t*-test (**p* < 0.05, ***p* < 0.01, ****p* < 0.001, NA, not available)*.

*ND, not detected*.

## Abbreviations

BBB, blood-brain barrier; CE-TOFMS, capillary electrophoresis with time-of-flight mass spectrometry; CNS, central nervous system; DA, dopamine; Ex-GF, ex-germ-free; GF, germ-free; MGB axis, microbiota-gut-brain axis; RSD, relative standard deviations.
